# Condensate Halos in Condensation Frosting

**DOI:** 10.1002/advs.202410657

**Published:** 2025-02-14

**Authors:** Songyuan Zhen, Haoyan Feng, Shiji Lin, Yakang Jin, Zhigang Li, Xu Deng, Elmar Bonaccurso, Longquan Chen

**Affiliations:** ^1^ School of Physics University of Electronic Science and Technology of China Chengdu 611731 P. R. China; ^2^ Department of Mechanical and Aerospace Engineering The Hong Kong University of Science and Technology Clear Water Bay Kowloon Hong Kong P. R. China; ^3^ Institute of Fundamental and Frontier Sciences University of Electronic Science and Technology of China Chengdu 610054 P. R. China; ^4^ AIRBUS Central R & T, Materials X 81663 Munich Germany

**Keywords:** condensate halo, condensation frosting, drop freezing, explosive evaporation, heat and mass transfer

## Abstract

The freezing of water drops on cold solid surfaces is ubiquitous in nature, and generally causes serious technological, engineering, and economic issues in industrial applications. Despite longstanding research efforts, existing knowledge on dropwise freezing is still limited, as this phase‐change phenomenon is always accompanied by complex heat and mass transfer processes. Herein, drop‐freezing phenomena in condensation frosting are investigated under standard laboratory conditions of humidity and pressure, highlighting their distinctions from those under some limiting conditions. Condensate halos consisting of massive tiny droplets are observed to form, grow, and eventually fade in a well‐defined region around freezing supercooled drops on sufficiently hydrophobic surfaces with low thermal conductivities. The detailed halo evolution is very different from that reported previously in ultradry and low ambient pressure environments, and it shows no identifiable effect on the long‐term frost propagation. By combining optical and thermal imaging techniques, this study scrutinizes the halo pattern evolution involving multiphase transitions on timescales from milliseconds to seconds, assesses the halo characteristics at each stage, and elucidates the underlying mechanisms. The work expands the fundamental understanding of complex dropwise freezing dynamics, and relevant findings can provide important guidance for developing anti‐icing/frosting strategies.

## Graphical abstract

The freezing of supercooled water drops on cold solid surfaces is an interfacial phase‐change phenomenon accompanied by complex heat and mass transfer yet remains partly understood. By combining optical and thermal imaging techniques, the study scrutinizes the formation, growth, and fading of condensate halos consisting of massive small droplets around freezing drops in condensation frosting, and uncovers the underlying mechanisms.

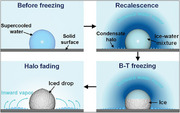

## 1 Introduction

Frosting and icing on cold solid surfaces are the most common phase change phenomena on our water‐covered Earth.^[^ ^1^ ^]^ In most cases, they bring about detrimental impacts on engineering applications, ranging from compromising power transmission efficiency^[^ ^2^ ^]^ to impairing aviation safety.^[^ ^3^ ^]^ These technological issues have motivated academic and industrial researchers to seek and develop strategies for frost/ice protection since long.^[^ ^4^, ^5^, ^6^, ^7^, ^8^ ^]^ Over the past few decades, there has been growing interest in manufacturing solid surfaces with anti‐icing/frosting functions, including water‐repellency at supercooled temperatures,^[^ ^9^, ^10^ ^]^ low ice adhesion strength,^[^ ^11^, ^12^, ^13^ ^]^ and delayed ice nucleation.^[^ ^14^, ^15^, ^16^ ^]^ Compared to conventional deicing techniques that manage to remove ice accretions by exploiting thermal heating, mechanical vibration, or air blowing,^[^ ^2^, ^3^, ^17^ ^]^ anti‐icing and anti‐frosting methodologies aim at reducing ice buildup on solid surfaces.^[^ ^18^ ^]^ To optimize these methodologies, a detailed understanding of the underlying mechanisms of ice nucleation, growth, and propagation is the necessary prerequisite for the design, fabrication, and effective use of anti‐icing/frosting surfaces.^[^ ^4^, ^10^, ^19^ ^]^

In humid environment, ice forms on cold solid surfaces through either desublimation or condensation frosting.^[^ ^1^, ^5^ ^]^ However, while desublimation—which transforms airborne water vapor directly into solid ice without becoming liquid in between^[^ ^20^ ^]^—takes preferentially place at atmospheric temperatures below − 10 °C, condensation frosting proceeds via a consecutive vapor‒liquid‒solid phase transition and is the most common icing mode in natural environment at temperatures just below 0 °C.^[^ ^15^, ^19^ ^]^ Condensation frosting begins with water vapor condensing into liquid water by forming microdrops on solid surfaces below the dew point.^[^ ^21^ ^]^ When the surface temperature decreases below the supercooling temperature, the condensed water drops start freezing. First, heterogeneous ice nucleation would be initiated at some nucleation sites on the solid‐liquid interface,^[^ ^22^, ^23^ ^]^ proceeding by growing ice dendrites within one of these supercooled drops; accordingly, the realsed latent heat of solidification heats the freezing drop up to ≈0 °C.^[^ ^5^, ^6^ ^]^ This is referred to as recalescence^[^ ^24^ ^]^ and followed by a slower freezing process in which the entire drop solidifies from its bottom to its top (B‐T freezing).^[^ ^25^ ^]^ The frozen drop then becomes the initiating site for ice propagation, which occurs by growing ice bridges toward neighboring drops that are still liquid and feed the ice bridges via their vapor.^[^ ^26^, ^27^ ^]^ The condensation frosting process ends when an interconnected ice network is built up across the entire solid surface and all liquid water is frozen. Despite extensive investigations,^[^ ^5^, ^6^ ^]^ several aspects of condensation frosting are still not clear, mainly because the phase transitions are accompanied by heat and mass transfers in an open system, which could induce additional phenomena influencing the icing/frosting processes. One intriguing discovery was that the latent heat released from a freezing drop can cause an “explosive” evaporation with outward vapor diffusion, establishing a local vapor field around the drop itself.^[^ ^28^ ^]^ If the environment is dry (relative humidity of <2%) or/and maintained at a pressure of a few mbar, the emitted water vapor will condense on the solid surface and form a halo of many small droplets surrounding the freezing drop, which can subsequently freeze into frost,^[^ ^29^, ^30^ ^]^ or it directly desublimates into ice embryos;^[^ ^31^ ^]^ both have been demonstrated to promote ice propagation among supercooled drop clusters on the surface. From an application point of view, we must therefore put forward the question whether these freezing‐induced heat and mass transfers also exist in condensation frosting phenomena under environmental conditions that are close to industrial manufacturing or transport operations, and if they should be considered in the design and fabrication of anti‐icing/frosting surfaces.

In this Article, we report observations of condensate halos around freezing supercooled water drops during condensation frosting on sufficiently hydrophobic surfaces with low thermal conductivities under standard laboratory conditions of humidity and pressure. We experimentally identified three stages of halo pattern evolution and elucidated the underlying mechanisms that occur on different timescales. The condensate halo extended to its full size around the freezing drop along with the short‐time recalescence, then continued to grow by condensing the emitted water vapor during the B‐T drop freezing, and eventually faded away after the drop completely froze to ice. Condensation halo evolution was found to occur within a well‐defined region around each freezing drop on timescales from milliseconds to seconds, which did not seem to affect the long‐term frost propagation on solid surfaces of different properties within the experimental conditions investigated.

## 2 Results and Discussion

### 2.1 Halo Pattern Formation, Growth, and Fading in Dropwise Condensation Frosting

**Figure 1:**
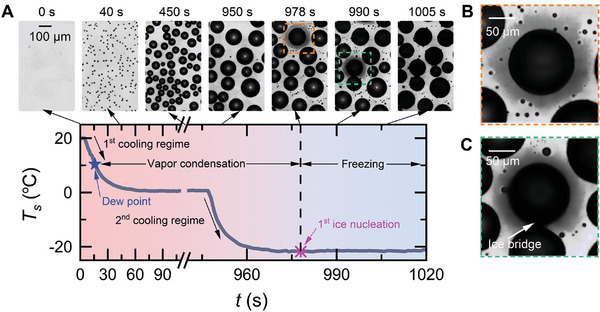
Representative images of an entire process of condensation frosting. A) A Teflon‐coated hydrophobic substrate was cooled from 20 °C down to − 20 °C through two steps; during these steps, dropwise condensation and dropwise freezing with halo pattern evolution were imaged using a top‐view camera (Movie S1, Supporting Information). B) Zoomed view showing a freezing drop that occurs spontaneously at 978 s. C) Zoomed view showing a freezing drop that occurs under the trigger of a contacting ice bridge at 990 s.

Condensation frosting experiments were carried out under standard atmospheric pressure (1 atm) in an environmental chamber; the initial relative humidity (χ  =  30% − 70%) inside the chamber was modulated by flushing with humid nitrogen with the desired gas ratio (Table S1, Supporting Information). Since the Péclet numbers for mass and heat transfer are always much smaller than one (Table S2, Supporting Information), the convection effect can be ignored in our experiments. Moreover, the radiation heat transfer is also generally negligible given that we performed all experiments in an environmental chamber.^[^ ^29^, ^31^ ^]^ Eighteen solid substrates (Table S3, Supporting Information) with different thermal conductivities (*k*  =  0.9 − 400 Wm^−1^K^−1^) and wetting properties (equilibrium water contact angles θ_*eq*_ =  30°−150°) were prepared and tested. In each trial, the solid substrate was first cooled from the ambient temperature (*T*_0_ =  20 ± 1 °C) to a defined temperature below the dew point (*T_d_* = 2−14°C, depending on χ^[^ ^32^ ^]^) of the surrounding gas, then maintained at this temperature for vapor condensation for a given time, and further cooled to the final temperature (*T_f_* = −2°C−25°C) for the freezing of condensed water drops (**Figure** **1**A). This stepwise substrate cooling enabled the observation of the freezing process of supercooled water drops of different sizes on diverse solid surfaces. The phase‐change phenomena on the cooling surface were monitored using a charge‐coupled device (CCD) camera from the top with a vertical illumination configuration (Section S1 and Figure S1, Supporting Information).

Figure 1A shows a whole condensation frosting process on a Teflon‐coated (with a thickness of 1 µm) glass substrate with *k* ≈ 0.9 Wm^−1^K^−1^ and θ_*eq*_ ≈ 120°, which was cooled to *T_f_* = −20°C through the stepwise method described above under χ ≈ 60%. Once the surface temperature *T_s_* was decreased to *T_d_* ≈ 10 °C, water vapor started to nucleate and grew into micrometer‐sized drops, which subsequently became supercooled when the temperature went below 0 °C. These condensate drops were initially transparent, and hence, their central regions appeared white due to the strong reflection and refraction of the incident light from the drop and solid surfaces (Section S2, Supporting Information). However, if ice nucleation was triggered, these drops immediately lost their transparency and “turned black” because of light refraction. This optical effect provides a useful tool for determining the onset of freezing and discriminating frozen from non‐frozen drops in optical images. Supercooled drops were found to freeze either spontaneously (Figure 1B) or when touched by ice bridges growing outward from neighboring frozen drops (Figure 1C).^[^ ^27^ ^]^ The former was more likely to develop in condensation frosting at 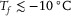, while the latter happened at all temperatures (Section S3, Supporting Information). Simultaneously with drop freezing, condensate halos consisting of a large number of small droplets of only a few micrometers in size formed and then disappeared in the very vicinity of the supercooled drops (Movie S1, Supporting Information), regardless of the freezing mode (Figure 1B,C). Note that these small droplets intercept light rays that are incident on and reflected from the solid surface; the larger and the denser the droplets are, the stronger the light interception is, and the darker the halo pattern would be.^[^ ^29^ ^]^ This halo pattern evolution could be clearly visualized on solid substrates with low thermal conductivities ($k≲10\;Wm^{-1}K^{-1}$) and poor surface‐wetting properties ($\theta_{eq}≳60^{\circ }$). A more hydrophobic or lower thermal conductivity of the surface correlated to a greater optical contrast between the halo pattern and the surface in the optical images (Section S4, Supporting Information). From the perspective of thermodyanmics, two necessary conditions are generally required to cause the condensate halo formation during drop freezing: establishing a supersaturated water vapor around the freezing drops (i.e., making the solid‐vapor system metastable) and overcoming the nucleation energy barrier (or equivalently the nucleus size should exceed a critical value).^[^ ^33^ ^]^ Practically, visualizing condensate halo patterns around freezing drops is only feasible if both the spatial and temporal resolutions of the optical imaging system are sufficiently high as they might be formed by very small droplets and last only for a very short time. Most recently, Shang et al. reported that there is a minimum freezing drop radius of ≈ 17.0 µm to trigger the formation of condensation halos.^[^ ^34^ ^]^ Within the spatial and temporal resolution limits of our optical imaging system, condensate halo evolutions were observed around freezing drops with radii down to ≈ 9.0 µm on the Teflon‐coated hydrophobic substrates (Section S4, Supporting Information). In the following, we will mainly discuss the experimental results on Teflon‐coated glass substrates; here, high‐contrast images of condensate halos were acquired, halo pattern characteristics were analyzed, and conclusions were drawn.

**Figure 2:**
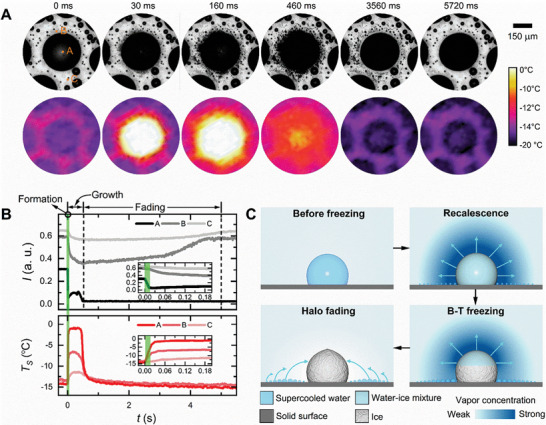
Dynamic evolution of the condensate halo around a freezing supercooled drop. A) Selected snapshots of the optical images (top) and simultaneously acquired thermographic images (bottom) showing the halo pattern evolution and the heat transfer landscape during the freezing of supercooled water drop at *T_f_* ≈ 14.5 °C and χ  =  60% on the Teflon‐coated hydrophobic surface in Figure 1 (Movie S3, Supporting Information). B) Temporal evolution of the pixel intensity *I* and surface temperature *T_S_* at the center of the freezing drop and two nearby locations denoted in (A). C) Cartoons depicting the vapor transport in different stages of drop freezing and halo pattern evolution.

To explore further aspects of the thermodynamic landscape of condensation frosting, we combined optical and thermal imaging techniques to simultaneously monitor the drop‐freezing process from the top (Figure S1 and Movie S2, Supporting Information). **Figure** **2**A displays a representative sequence of halo dynamics and the associated temperature evolution during the spontaneous freezing of a supercooled condensate drop at *T_f_* = −14.5 °C and χ ≈ 60% (Movie S3, Supporting Information). We can identify three distinct stages of heat and mass transfer at different timescales. Similar results have also been obtained for freezing drops triggered by a contacting ice bridge (Section S5 and Movie S4, Supporting Information). Upon freezing (indicated by the sudden decrease in the pixel intensity *I* in the optical image—top panel in Figure 2B), the drop temperature immediately increases from *T_f_* to the melting point of ice (*T_m_* = 0 °C, bottom panel in Figure 2B) due to recalescence.^[^ ^20^, ^24^ ^]^ Recalescent freezing is a kinetic crystallization process during which a dendritic ice network forms in the supercooled drop,^[^ ^35^ ^]^ turning it into a mixture of liquid water and ice crystals. The latent heat released from the phase change causes the sharp temperature increase of the drop and by heat transfer also of its nearby region (bottom panel of Figure 2B). With drop temperature rising, the saturation vapor pressure of the ice/water mixture also increases. With increasing vapor pressure, more water molecules evaporate from the drop surface and diffuse into the surrounding space, generating a warmer radiant vapor field (shown in Figure 2C). Moreover, water vapor in the supersaturated region close to the cold surface condenses into liquid water, forming a halo‐like pattern around the recalescing drop (Figures 1 and 2A). These multiple phase transitions happened in a flash, but still on a detectable timescale of a few to tens of milliseconds (light green regions in Figure 2B). Thereafter, drop freezing enters the process of solidifying the remaining liquid water among ice crystals, which starts at the drop‒solid interface. The solidification front gradually moves upward to the top of the drop,^[^ ^25^ ^]^ typically lasting for hundreds of milliseconds for submillimeter‐sized drops. During such B‐T freezing, the upper part of the drop remains a mixture of water and ice at *T_m_* ≈ 0°C. This temperature is still higher than that of the cold substrate and of the surrounding atmosphere (30 ms in Figure 2A). Therefore, the warm, humid vapor field persists around the freezing drop (sketched in Figure 2C), and vapor continues to condense on the cold surface around the drop in the form of microdrops. As a consequence, the as‐formed condensate microdrops in the halo region grow in size—either individually or by coalescing with their neighbors^[^ ^21^ ^]^—leading to a darker halo pattern appearance (30−460 ms in Figure 2B). When the drop is completely frozen, the source of latent heat expires, and its temperature and the temperature of the halo region quickly decrease to *T_f_*. Due to the very low vapor pressure over ice,^[^ ^36^ ^]^ the frozen drop then becomes a vapor sink and starts harvesting water molecules from the water droplets of the halo through the well‐known Wegener–Bergeron–Findeisen (WBF) process:^[^ ^27^, ^37^ ^]^ by the vapor pressure gradient between supercooled condensate microdrops of the halo and the frozen drop, the microdrops evaporate, and their vapor is directly deposited on the frozen drop, contributing to its growth. The halo pattern gradually fades away from the inner to the outer perimeter (460−5720 ms in Figure 2A). Although these condensate drops are very small, the halo fading process takes a few tens of seconds, which is typically one to two orders of magnitude longer than the formation and growth of the condensate halo. The different durations of the three stages of halo pattern evolution (formation, growth, and fading) indicate that their governing dynamics are distinct and will be separately analyzed in the following sections.

### 2.2 Halo Formation in Recalescent Freezing

**Figure 3:**
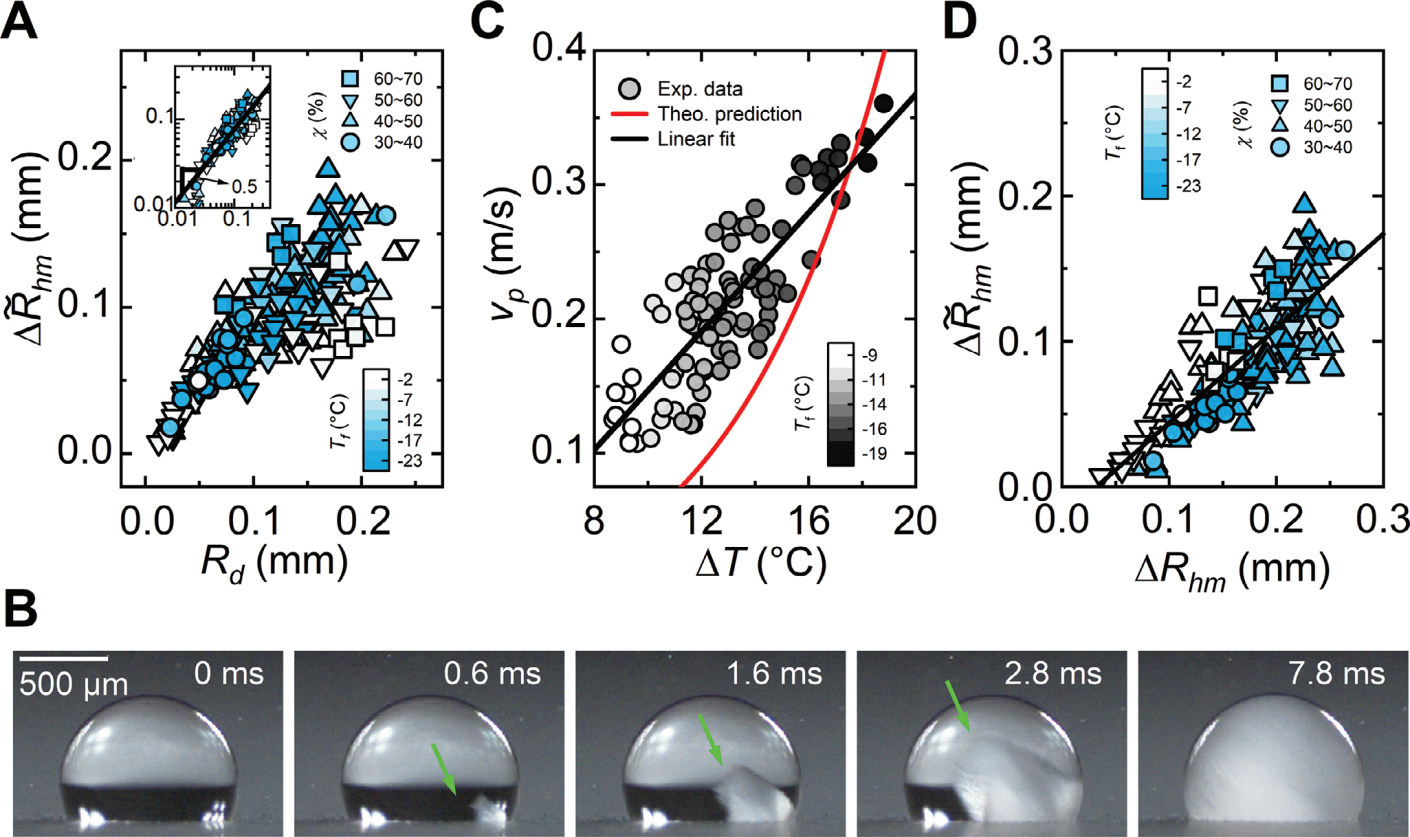
Characteristic size and duration of condensate halo formation during recalescent freezing. A) Equivalent halo radial extension $\Delta {\tilde{R}}_{hm}$ versus drop radius *R_d_*. The inset is the log‐log plot of the same data. B) Selected optical images showing the growth of dendritic ice crystals in a 3.3 µL supercooled water drop at *T_f_* = − 13.2 °C and χ  =  55% (Movie S5, Supporting Information). C) Characteristic velocity of ice propagation *V_P_* versus the supercooling temperature Δ*T*. D) Plot of the halo radial extension $\Delta {\tilde{R}}_{hm}$ measured in the experiment versus the halo radial extension Δ*R_hm_* predicted by the theoretical model.

Drop freezing starts with recalescence, whereby the supercooled drop is partially solidified by rapidly growing dendritic ice crystals inside^[^ ^20^, ^38^ ^]^ and thus becomes opaque (Figure 1A). The simultaneous decrease of the pixel intensity values of the freezing drop and its nearby region in optical images (Figure 2A) indicates that vapor condensation occurs almost coincidently with the recalescent freezing, forming a halo‐like pattern of microdrops all around it. More strikingly, the maximum halo extension is instantly attained upon freezing (Figure 2B), i.e., at a timescale smaller than our highest resolution. This observation contrasts with that of freezing drops under low humidity, where condensate halos were found to appear only after the occurrence of recalescence.^[^ ^29^, ^30^ ^]^ The short‐time dynamics of halo formation can be attributed not only to the rapid buildup of the vapor field via the explosion‐like evaporation and diffusion of water vapor, whose characteristic time is *l* ^2^/*D_v_* ≈ 0.4 ms, but also to the rapid heating of the surrounding atmosphere; this increases both the partial pressure of water vapor^[^ ^32^ ^]^ and the water condensation rate. Here, *l* is the characteristic length, which is on the order of the drop radius, and *D_v_* is the diffusion coefficeint of water molecules in the vapor phase. The role of a warmer temperature field in halo formation can be indirectly inferred from similar measurements on hydrophobic substrates with high thermal conductivities. On them, the formation of condensate halos when supercooled drops freeze is much less extended—or even suppressed—and the surrounding temperature field is colder (Section S6, Supporting Information) due to the faster heat conduction away from the heat source of the drop. The characteristic time for heat transfer through solid substrates can be estimated as *h* ^2^/*α*, which is typically on the order of 10 ms and comparatively shorter than the time of drop recalescence on solid substrates with $k≲10\;Wm^{-1}K^{-1}$. Here, *h* denotes the thickness of the substrates and *α* is the thermal diffusivity. Once fully extended, we characterized the halo pattern by measuring the equivalent radial extension $\Delta {\tilde{R}}_{hm}$ for its irregular perimeter, and the results for all condensate halos are plotted as a function of the radius of the freezing drop *R_d_* in **Figure** **3**A. Note that *R_d_* is the radius of the drop equator. $\Delta {\tilde{R}}_{hm}$ is on the same order of magnitude as *R_d_*, and nonlinear positive correlations are observed regardless of the freezing temperature and initial relative humidity. A log‐log plot of the same data shows that $\Delta {\tilde{R}}_{hm}$ increases with increasing *R_d_* following a one‐half power law (i.e., ${\tilde{R}}_{\mathrm{hm}}\;∼R_{d}^{1/2}$, inset of Figure 3A) over at least one order of magnitude of the radii from ≈0.01 to ≈0.1 mm.

To gain more insight into the halo formation mechanism, the duration of the recalescence process needs to be first examined. Because of their small sizes and their accumulation in clusters (Figure 1A), the crystal growth inside freezing drops cannot be visualized during condensation frosting; instead, we studied the recalescent freezing of individual submillimeter‐ to millimeter‐sized water drops under different supercooling temperatures by high‐speed imaging. Once heterogeneous ice nucleation is triggered—in most cases at some locations on the solid‐liquid interface—it is accompanied by rapidly growing crystalline dendritic branches inside the drop oriented toward different directions (Figure 3B; Movie S5, Supporting Information). The time *t_rf_* required for the dendritic ice to propagate across the entire drop provides a measure of the recalescent freezing process. *t_rf_* was determined to be ∼5−∼21ms for freezing drops of radii *R_d_* =  0.5 − 1.1 mm, being shorter at higher supercooling temperatures Δ*T*  = *T_m_*  − *T_f_* (Figure 3C). The dendritic crystal growth is the most common form of liquid solidification^[^ ^38^ ^]^ and the steady‐state motion of the dendritic tip can be described by^[^ ^39^ ^]^ $\Delta T\cdot c_{p}/L=Pe\cdot e^{Pe}\int_{Pe}^{\infty }\frac{e^{-\xi }}{\xi }d\xi$. Here, *c_p_* and *L* are the specific heat capacity and the latent heat of the melting phase, respectively; *Pe*  = *r_t_* *V*/2*α*_*m*_ is the Peclet number; *r_t_* is the radius of the growing ice crystal tip; *V* is the tip velocity and *α*_*m*_ is the thermal diffusivity of the melting phase. The steady‐state crystallization theory predicts a velocity of the order of 0.1 m s^−1^ and that *V* increases with increasing degree of supercooling in our experimental temperature range of 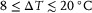 (red solid line in Figure 3C). Due to the difficulty in measuring the velocity of dendritic ice growing in multiple directions (Figure 3B), we performed a semiquantitative analysis by defining and calculating a characteristic velocity for ice propagation on different freezing drops *V_P_* = π*R_d_*/*t_rf_*. Good agreement between the theoretical prediction and the experimental observation was reached on the magnitude of the crystallization velocity (Figure 3C). However, rather than following the polynomial trend suggested by the theory, the experimental data could be effectively fitted by a linear correlation, *V_P_* = Δ*T*/*A*, or:

(1) $$\;t_{rf}=\frac{\pi AR_{d}}{\Delta T}$$

where *A* is a coefficient determined to be ≈0.02 K · s m^−1^. This discrepancy can be ascribed to the fact that the theoretical model was derived for the tip velocity of an isolated growing dendrite,^[^ ^39^ ^]^ while the effective velocity for dendritic ice growth in various directions was determined from our experiments.

To understand the power‐law relationship between the extension of condensate halos and the radii of the freezing drops they surround (Figure 3A), we theoretically looked at the rapid halo formation process. Upon recalescent freezing, the sudden heating of the supercooled drop and its nearby region induces a parallel increase in the water vapor pressure above the drop and thus to the transient water vaporization. Water vapor diffuses quickly due to the steep vapor gradient around the freezing drop and condenses into microdrops when it gets into contact with the surface cooled below the dew point. This dropwise condensation has the pattern of a halo. Although these heat and mass transfer processes occur simultaneously, or at least partially overlap, vapor diffusion, which directly determines the water saturation level in the ambient environment of the freezing drop, should dominate halo formation. The geometric symmetry of the freezing drop and its surrounding space allows the modeling of the transient water vapor diffusion with the 1D form of Fick's second diffusion law: $\frac{\partial C}{\partial t}=D_{v}\;\frac{\partial^{2}C}{\partial x^{2}}$, where *C* is the water vapor concentration, and *x* is the distance from the vapor source (i.e., the freezing drop) with *x*  =  0 located at the drop surface. Under the initial condition, *C*  = *C*_0_ at *t*  =  0 for *x* > 0 and the boundary condition, *C*  = *C_d_* at *x*  =  0 for *t* > 0, the general solution of the diffusion equation is as follows:^[^ ^40^ ^]^ $\frac{C-C_{d}}{C_{0}-C_{d}}=1-\mathrm{erfc}\left(Z\right)$, where $\mathrm{erfc}\left(Z\right)=\frac{2}{\sqrt{\pi }}\int_{0}^{z}e^{-\xi^{2}}d\xi$ is the complementary error function with $Z=\frac{x}{2\sqrt{D_{v}t}}$. Considering that *Z* ≪ 1 for vapor diffusion at $x\leq \Delta {\tilde{R}}_{hm}\approx O\left(0.1\;\mathrm{mm}\right)$ during recalescent freezing [*t_rf_* ≈ *O*(10 *ms*)], the spatial‐temporal evolution of the vapor field can be written as $C\approx C_{0}-\frac{(C_{0}-C_{d})x}{\sqrt{\pi D_{v}t}}$. Condensate halos are presumed to develop on the cold surface region, above which the water concentration is higher than or at least equal to its saturation value *C_S_*. One can then derive the formula for the maximum radial extension:

(2) $$\Delta R_{hm}\approx \frac{C_{S}-C_{0}}{C_{d}-C_{0}}\sqrt{\pi D_{v}t_{rf}}\approx \pi \frac{C_{S}-C_{0}}{C_{d}-C_{0}}\sqrt{\frac{AD_{v}R_{d}}{\Delta T}}$$

The above equation predicts a scaling correlation $\Delta R_{\mathrm{hm}}∼R_{d}^{1/2}$, which matches well with the experimental observation in Figure 3A. The rationality of the model can be further examined by a direct comparison between the experimental and theoretical results, where a linear correlation is expected and indeed obtained Figure 3D.

### 2.3 Halo Growth During Bottom‐to‐Top Drop Freezing

**Figure 4:**
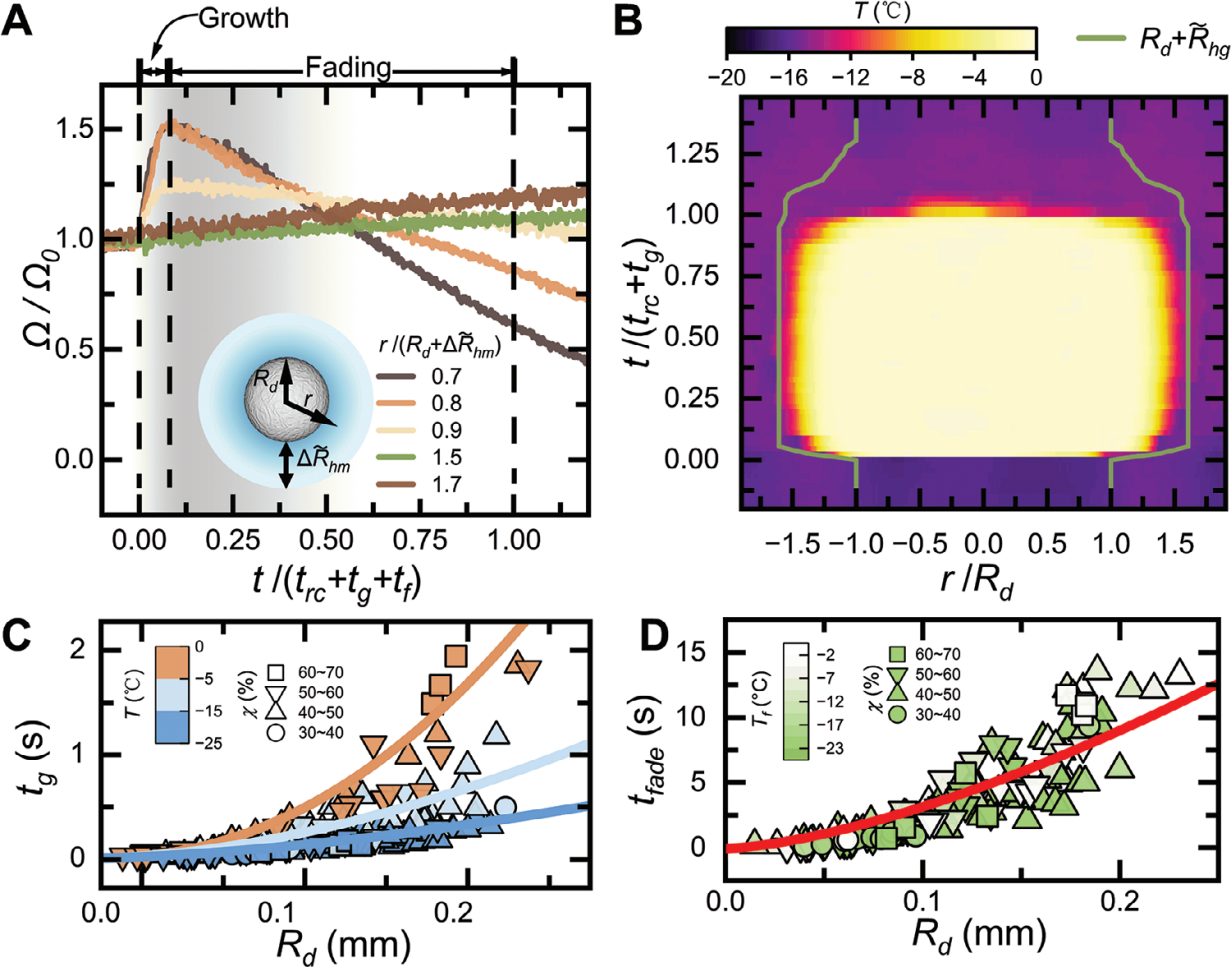
Characterization of the halo growth and fading processes. A) Normalized volume Ω/Ω_0_ versus time *t*/(*t_rc_* + *t_g_* + *t_fade_*) of five as‐formed condensate microdrops at different locations in Figure 2A during the halo growth and fading processes. B) Spatiotemporal evolution of the temperature distribution along the horizontal central line on the freezing drop in Figure 2A. The solid line denotes the farthest location of halo growth region $r=R_{d}+\Delta {\tilde{R}}_{hg}$ with $\Delta {\tilde{R}}_{hg}$ the equivalent size of the halo growth. C) Growth time of the condensate halos (*t_g_*) versus the radii (*R_d_*) of the freezing drops that the halos surround under different experimental conditions. d) Fading times of condensate halos *t_fade_* versus the radii (*R_d_*) of the freezing drops that the halos surround under different experimental conditions.

The extended condensate halos during recalescent freezing are quite faint (see 30 ms in Figure 2A). Less than 20% of the supercooled drop forms ice crystals,^[^ ^41^ ^]^ while the remaining liquid water freezes in a second stage that is much slower and proceeds from the bottom of the drop to its top.^[^ ^25^ ^]^ In this stage, water continues evaporating from the partially frozen drop and diffuses radially outward, serving as the vapor source feeding the halo pattern via condensation; it causes the growth of condensate droplets in the halo region (30−460 ms in Figure 2A). Since the diffusive timescale is typically orders of magnitude shorter than the B‐T freezing time, the resulting vapor field around the freezing drop can thus be considered in a quasi‐steady state, obeying the Laplace equation ∇^2^ *C*  =  0.^[^ ^29^ ^]^ Notably, the B‐T drop solidification reduces the volume and surface area of the liquid water and accordingly the saturated vapor field above the iced surface. Thus, a reduction in the halo growth region with ongoing freezing is intuitively expected (Section S7, Supporting Information). However, our experimental observations show that vapor condensation is constant in the initially formed halo region (i.e., in the region with radial extent $\Delta {\tilde{R}}_{hm}$), where the pixel intensity values in the optical images continuously decrease (Figure 2B). More direct evidence was provided by tracking the volume variations in the as‐formed, measurable condensate drops prior to halo formation during the B‐T freezing. As displayed in **Figure** **4**A, immediately following recalescence, the growth rate of those condensate drops increased in the halo region with $r/(R_{d}+\Delta {\tilde{R}}_{hm})≲1$, while a slow but steady drop growth was identified at $r/(R_{d}+\Delta {\tilde{R}}_{hm})≲1$. These findings suggest the local nature of the drop‐freezing‐induced vapor field: it only affects the heat and mass transfer in a well‐defined region, beyond which the background vapor concentration of the environmental chamber predominates. As a result, condensate halo evolution during B‐T drop freezing proceeds by enlarging the volumes of all condensate droplets in the same halo region developed during recalescent freezing, and not elsewhere.

The observed nontrivial halo growth could be attributed to the established vapor field and temperature field around the freezing drop, and their coupling effects; the former ensures a sufficiently high water molecule concentration in the environment above the cooling surface, while the latter reduces the dew point for vapor condensation.^[^ ^32^ ^]^ Figure 4B compares the spatiotemporal evolution of the temperature distribution and the determined halo growth region (where $r=R_{d}+\Delta {\tilde{R}}_{hg}$ denotes the farthest location of halo growth region with ${\tilde{\Delta R}}_{hg}$ being the equivalent size of the halo growth region, see methods) along the horizontal central line on the freezing drop in Figure 2A. Condensate halo growth only occurred in the vapor field where vapor temperature is sufficiently high (≈ 3 °C higher than the solid surface temperature). Nevertheless, the time‐dependent vapor field around the freezing drop does affect the vapor condensation rate, which decreases with increasing the freezing time or the distance from the freezing drop (Figures 2B and 4A). Eventually, the well‐developed halo patterns consist of nonuniformly distributed microdrops, with sizes and number densities decreasing radially outward from the B‐T freezing mother drop (Figure 2C), which can be observed in Figure 2A and is also evidenced by the radial increase of the pixel intensity values in the outward direction (Figure S2E, Supporting Information).

The halo growth stops when the drop is completely frozen, as indicated by the optical intensity attaining the lowest level and the surface temperature decreasing to *T_f_* (Figure 2B). Figure 4C summarizes the growth time (*t_g_*) for condensate halos formed around freezing drops under different experimental conditions of temperature and initial relative humidity. *t_g_* nonlinearly increases with increasing *R_d_* or *T_f_*, while no dependence of *t_g_* on χ was observed. Given that the halo growth occurs simultaneously with B‒T drop freezing, its duration should be the same as the freezing time. Since the B‒T freezing of clusters of microdrops is difficult to track experimentally, we instead performed a theoretical analysis on this typical Stefan‐type problem with a moving interfacial boundary. Our experimental observations have demonstrated that the slushy top of the freezing drop and its nearby region generally maintain at the same temperature (≈ 0 °C) throughout the freezing process (Figure 2B); thus, this process can be considered isothermal. The heat transfer on the freezing drop can be described this way:^[^ ^42^ ^]^ the latent heat released from the advancing solidification front per unit time, ${\dot{Q}}_{L}=\rho_{m}L_{m}\left(dH_{f}/\mathrm{dt}\right)S_{f}$, is conducted mainly through the frozen part underneath to the cooling substrate at a rate, ${\dot{Q}}_{C}=k_{i}\nabla TS_{f}$. Here, *ρ*_*m*_ and *L_m_* are the density and latent heat, respectively, of the water‐ice mixture; *H_f_* and *S_f_* are the height and surface area, respectively, of the solidification front; *k_i_* represents the thermal conductivity of the ice; and ∇*T* denotes the temperature gradient in the solidified part. Balancing these two terms of the heat flux rate and further assuming a linear temperature gradient in the frozen drop bottom (i.e., ∇*T* ≈ Δ*T*/*H_f_* as first‐order approximation) we can write the duration of the halo growth as follows:

(3) $$t_{g}\approx \;\frac{\rho_{m}L_{m}H_{d}^{2}}{2k_{i}\Delta T}=\frac{\rho_{m}L_{m}{\left(1-\cos\theta_{eq}\right)}^{2}}{2k_{i}\Delta T}\;R_{d}^{2}$$

where *H_d_* = *R_d_* (1 − cos θ_*eq*_) is the drop height. Equation (3) suggests a square‐law correlation between *t_g_* and *R_d_*, and the prefactor is inversely proportional to the degree of supercooling, agreeing well with the experimental data in Figure 4C.

### 2.4 Halo Fading After Drop Freezing

Once the drop is completely frozen, its role in the vapor field changes from a source to a sink. Then, the frozen drop directly retrieves water molecules from the nearby still liquid condensate droplets, accelerating their evaporation. The temporal evolution of the condensate drop volumes in Figure 4A implies that this reversal of mass transfer is also confined within the halo region; the closer to the frozen drop the condensate droplet is, the faster its evaporation is. As a result, condensate halo patterns gradually disappear from the inside to the outside (Figure 2A). This halo fading behavior is different from that observed during the freezing of supercooled drops under very low humidity, as reported in Ref. [29] where water vapor from evaporating halos mostly diffuses into the surrounding dry environment and thus follows an outside‐to‐inside fading route. In Figure 4D, we plot the total fading time *t_fade_* for different condensate halos formed around freezing drops of different sizes. Briefly, *t_fade_* nonlinearly increases from ≈ 31 ms for freezing drops with *R_d_* ≈ 12 µm to ≈ 13.2 s for freezing drops with *R_d_* ≈ 230 µm, regardless of the other experimental conditions.

The observed halo fading process is complex, not only because it involves liquid evaporation and vapor sublimation‐condensation but also because these processes simultaneously occur among a large number of supercooled drops, where coupled heat and mass transfers takes place.^[^ ^43^, ^44^ ^]^ We present here a simple rationale to explain the nonlinear dependence of the halo fading time on the freezing drop radius. Notwithstanding their simultaneous evaporation, condensate droplets in the halo region fade away in a well‐defined sequence from the nearest to the farthest (Figure 2A; Movie S3, Supporting Information). Based on this and the directional deposition of water vapor, we propose that a condensate droplet at a distance *l* from the frozen drop completely dries when all water molecules are transported to the frozen drop by diffusion; thus, its characteristic evaporation time is τ_*e*_ = *l* ^2^ /*D_v_*. Although this scaling analysis is simple, it provides a proper estimation of the timescale of droplet evaporation during halo fading and reasonably accounts for the effect of the spatial distribution of condensate droplets on their evaporation. Furthermore, assuming that condensate droplets are located on a radial lattice with an average number density ${\bar{n}}_{d}$, the fading time of the entire halo pattern should be on the order of the sum of the evaporation times of all individual droplets: $t_{fade}∼\int_{0}^{R_{hm}}\tau_{e}{\bar{n}}_{d}dl∼R_{d}^{3/2}$. As shown in Figure 4D, the experimental data can be effectively fitted using a power law correlation $t_{fade}=aR_{d}^{3/2}$, with the coefficient *a* ≈ 100 s · mm^−3/2^.

### 2.5 Effects of Halo Dynamics on Frost Propagation Over Surfaces

**Figure 5:**
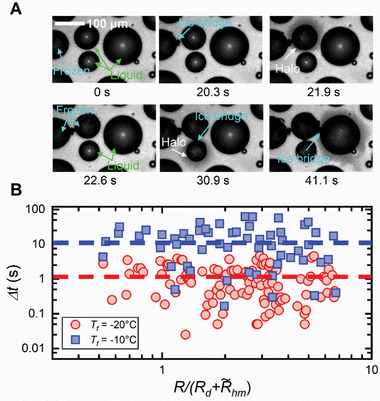
Frost propagation during condensation frosting. A) Sequence of images showing the ice‐bridge‐assisted frost propagation among the supercooled condensate drops at *T_f_* = − 14.4°C and χ  =  65% (Movie S6, Supporting Information). B) Time interval Δ*t* of two sequential freezing events without developing the ice bridge versus the normalized distance between these two drops $R/(R_{d}+\Delta {\tilde{R}}_{hm})$ in condensation frosting occurring at *T_f_* = − 20 °C and χ  =  60%. The dashed lines indicate the average times.

Finally, we clarify whether the described halo formation, growth and fading processes affect the long‐term condensation frosting phenomenon on solid surfaces. The vapor emitted from freezing supercooled water drops is known to induce domino‐like freezing among drop clusters under dry or/and low‐pressure environmental conditions via two distinct processes: the formation of a condensate halo that further freezes into frost and contacts its neighboring drops^[^ ^29^ ^]^ or the formation of airborne ice crystals in the diffusive vapor when the emitted vapor reaches nearby supercooled drops.^[^ ^31^ ^]^ Although direct contact freezing is the main frost propagation mode in our experiments (Figure S3, Supporting Information), condensate halos always fade away before freezing into frost. Generally, after halo fading, ice bridges grow from the frozen drop toward neighboring supercooled drops, which first evaporate and subsequently freeze upon contact with an ice bridge. A typical frost propagation among four supercooled drops is shown in **Figure** **5**A (Movie S6, Supporting Information). For other freezing events (i.e., drop freezing without connecting with a visible ice bridge), we measured the time intervals Δ*t* between two sequential freezing drops, and the results for condensation frosting at two different freezing temperatures are plotted as a function of the normalized distance $R/(R_{d}+\Delta {\tilde{R}}_{hm})$ (Figure 5B), where *R* denotes the distance between the two successively freezing drops. Δ*t* is shorter at lower temperatures than at higher temperatures, and it spans a similar range of values for drops in the halo region [i.e., $R/(R_{d}+\Delta {\tilde{R}}_{hm})\leq 1$] and those beyond [i.e., $R/(R_{d}+\Delta {\tilde{R}}_{hm})>1$]. This finding rules out the possibility of directly forming ice crystals from the freezing‐induced water vapor (i.e., desublimation), which can only happen in very dry atmosphere or at low ambient pressure. We conclude from our measurements that condensate halos that form around freezing drops during condensation frosting in an ambient atmospheric environment do not bring additional effects on the subsequent drop cluster freezing and thus on frost propagation on solid surfaces. This stands in contrast to what happens in more extreme environmental conditions.^[^ ^29^, ^31^ ^]^

## 3 Discussion

We have shown that the formation of condensed microdrop halos, which was reported to occur during the freezing of supercooled sessile water drops in ultradry environments (relative humidity χ ≤ 2%),^[^ ^29^, ^31^ ^]^ does also take place in higher ambient humidity (χ = 30%−70%). The halo was best developed and visible on sufficiently hydrophobic surfaces ($\theta_{eq}≳60^{\circ }$) with low thermal conductivities ($k≲10\;Wm^{-1}K^{-1}$), but to different extents it formed on surfaces with any wettability or thermal conductivity. The formation, growth and fading (i.e., condensation and evaporation) of microdrop halos during condensation frosting involved multiple phase transitions and exhibited complex dynamic behaviors over a wide range of timescales, spanning three orders of magnitude. During the recalescence process of *O*(10 ms), a warm vapor was generated by the freezing drop and condensed on the cold solid surface all around it into a large number of small water droplets. In this stage, an embryonic but spatially fully extended halo pattern was formed. During the subsequent bottom‐to‐top drop freezing of *O*(100 ms), more water vapor from the partially frozen drop condensed onto the as‐formed small droplets, increasing the volume of liquid water in the halo pattern, but not its spatial extension. This two‐stage halo formation process is different from that observed earlier in a dry environment, where condensate halos were found to form only after recalescence.^[^ ^29^ ^]^ Within a few to tens of seconds [*O*(10 s)] after drop freezing, the small droplets of the halo completely evaporate because the vapor is recaptured by the completely frozen drop, above which the vapor pressure is nearly zero.

Our results reveal the complexity and richness of ice and frost formation phenomena, which are per se fundamental thermodynamic processes. Recognizing that new details of dropwise freezing phenomena were uncovered, we envision that our work can stimulate further experimental and theoretical research in the near future. Furthermore and to our disappointment, the identified condensate halo dynamics—despite being very robust and reproducible—does not affect the remaining supercooled drops and thus the frost propagation over the solid surface in ambient conditions. We were thus not able to propose strategies how to combat surface frosting in industrial processes under real operating conditions of pressure and temperature by simple passive means, i.e., by tuning the surface properties like wettability or the thermal properties of the substrate. Nevertheless, we believe that the condensate halo phenomenon is not a factor to consider when developing and optimizing anti‐icing/frosting strategies in diverse industrial processes.

## 4 Experimental Section

### Surface Preparation and Characterization

Eighteen solid surfaces with different thermal conductivities and wetting properties have been prepared. Smooth glass (*k* ≈ 0.93 Wm^−1^K^−1^), stainless steel (*k* ≈ 10 Wm^−1^K^−1^), silicon (*k* ≈ 160 Wm^−1^K^−1^), and copper (*k* ≈ 400 Wm^−1^K^−1^) substrates with thicknesses of 0.5 mm were successively cleaned with isopropanol, acetone, and ethanol in an ultrasonic bath for 10 min each, and used as hydrophilic surfaces (with θ_*eq*_ ≈ 31°, 80°, 60°, and 76°, respectively) after drying under nitrogen blowing. By treating clean glass and copper substrates with the oxygen plasma (plasma cleaner PDC‐002, Harrick Plasma) for 5 min and then immediately silanizing in the vapor phase of 3‐aminopropyltriethoxysilane (99%, Sigma–Aldrich, Merck) in a desiccator at 100 °C for 10 h, two other types of hydrophilic surfaces (θ_*eq*_ ≈ 62°) with different thermal conductivities were obtained. Employing the same silanization procedure to treat these four types of solid substrates with 1,1,1,3,3,3‐Hexamethyldisilazane (99.9%, Sigma–Aldrich, Merck) and 1H,1H,2H,2H‐perfluorodecyltriethoxysilane (98%, Sigma–Aldrich, Merck) further result in more hydrophobic surfaces with θ_*eq*_ ≈ 87 ± 1° and 103−117°, respectively. One more type of hydrophobic surfaces with θ_*eq*_ ≈ 120° were prepared by coating glass substrates with 1 µm thickness Teflon. Furthermore, superhydrophobic surfaces with θ_*eq*_ ≈ 150−152° were also fabricated by spraying these four solid substrates with a layer of 2 µm thick hydrophobic silica nanoparticles (Glaco Mirror Coat “Zero” from Soft 99 Co.). The roughness of all prepared surfaces were examined by a high‐resolution AFM (Bioscope Resolve, Bruker, USA) in an image area of 10 µm × 10 µm (Table S4, Supporting Information), while their wetting properties were characterized by measuring the equilibrium contact angles of 4 µL sessile pure water drops (18.4 MΩ cm, Millipore Synergy, Germany) using a commercial goniometer (OCA15LHT­‐SV, Dataphysics, Germany).

### Optical and Thermographic Image Acquiring and Processing

Condensation frosting processes on diverse solid surfaces under different experimental conditions were recorded using a CCD camera (U3‐3060CP‐M‐GL, IDS Scheer, Germany) and an infrared camera (A655sc, FILR, USA). The recalescent freezing of submillimeter‐ to millimeter‐sized water drops under different supercooling temperatures were captured by a high‐speed CCD camera (Phantom V2012, USA) at 5000 fps. The acquired black–white movies of condensation frosting events were processed using a custom‐programmed MATLAB (MathWorks, Inc.) algorithm. The temporal evolutions of the pixel intensity values in Figure 2 and in Figure S5 (Supporting Information) were obtained by calculating the average grayscale intensity of a circular region of 5 pixels at different locations on/near the freezing drops in the acquired images. The radii of condensate drops were determined by $R_{d}=\sqrt{A_{d}/\pi }$ with *A_d_* being the area of the drop, and the volumes of condensate drops in Figure 4A were computed by $\Omega =\frac{\pi }{3}R_{d}^{3}{\left(1-\cos\theta_{\mathrm{eq}}\right)}^{2}\left(2+\cos\theta_{\mathrm{eq}}\right)$. By tracing the variation of the pixel intensity values on the solid surface beyond the freezing drop during the recalescent freezing and B‐T freezing, irregular perimeters (e.g., the blue line in Figure 4B) that define the halo formation and growth regions were obtained, and the equivalent radial extent of halo pattern and the effective size of the halo growth region can be determined by $\Delta {\tilde{R}}_{hm}=\sqrt{A_{hm}/\pi }\;-R_{d}$ and $\Delta {\tilde{R}}_{\mathrm{hg}}=\sqrt{A_{\mathrm{hg}}/\pi }\;-R_{d}$, where *A_hm_* and *A_hg_* are the surface areas surrounded by the identified perimeters during halo formation and growth, respectively. The temperature information of freezing drops over time were directly extracted from the acquired thermographic images using the FLIR software.

### Statistical Analysis

To ensure the repeatability and reliability of the results, massive experimental measurements were performed and analyzed. More specifically, the characteristic sizes and durations of condensate halos in Figures 3A,D and 4C,D were obtained from 200 freezing drops in condensation frosting; the characteristic velocities of ice propagation in Figure 3C were acquired from the freezing of 100 sub‐millimeter and millimeter‐sized drops, while the time interval of two sequential freezing events in Figure 5B was determined from 130 freezing drops in condensation frosting.

## Conflict of Interest

The authors declare no conflict of interest.

## Author Contributions

L.C. and E.B. conceived and supervised the research. H.F. initiated the experiment and finished all measurements with S.Z., and they both analyzed the data. L.C. developed the analytic models, while S.Z. and H.F. validated relevant models with the experimental results. S.L. performed the numerical simulations. Y.J., Z.L., X.D., E.B., and L.C. interpreted the data. L.C. and E.B. wrote the manuscript with input from all authors. S.Z. and H.F. contributed equally to this work.

## Supporting information



Supporting Information

Supplemental Movie 1

Supplemental Movie 2

Supplemental Movie 3

Supplemental Movie 4

Supplemental Movie 5

Supplemental Movie 6

## Data Availability

The data that support the findings of this study are available from the corresponding author upon reasonable request.
