# Supplementary material for: Condensate Halos in Condensation Frosting
Source: Adv Sci (Weinh). 2025 Feb 14;12(14):2410657. doi: 10.1002/advs.202410657 (PMC11984898; doi:10.1002/advs.202410657)
Supplement: Supplementary file 1 — Supporting Information [file ADVS-12-2410657-s005.docx]

Supporting Information

**Condensate Halos in Condensation Frosting**

*Songyuan Zhen*^†^*, Haoyan Feng*^†^*, Shiji Lin, Yakang Jin, Zhigang Li, Xu Deng, Elmar Bonaccurso^*^ and Longquan Chen^*^*

S. Zhen, H. Feng, Dr. Y. Jin, Prof. L. Chen

School of Physics, University of Electronic Science and Technology of China, Chengdu 611731, P. R. China

Email: [lqchen@uestc.edu.cn](mailto:lqchen@uestc.edu.cn)

Dr. S. Lin, Prof. Z. Li

Department of Mechanical and Aerospace Engineering, The Hong Kong University of Science and Technology, Clear Water Bay, Kowloon, Hong Kong

Prof. X. Deng

Institute of Fundamental and Frontier Sciences, University of Electronic Science and Technology of China, Chengdu 610054, P. R. China

Dr. E. Bonaccurso

AIRBUS Central R & T, Materials X, Munich 81663, Germany

Email: [elmar.bonaccurso@airbus.com](mailto:elmar.bonaccurso@airbus.com)

^†^S.Z. and H.F. contributed equally to this work.

**Keywords**: condensate halo, drop freezing, condensation frosting, explosive evaporation, heat and mass transfer

# Supplementary Section 1. Experimental setup and procedures

We experimentally investigated condensation frosting phenomena on solid surfaces with different thermal conductivities and wetting properties (Table S1). The experimental setup is schematically illustrated in Figure S1. The environmental chamber (with inner dimensions of $10 cm\times10 cm\times4 cm$) was made of double walls of acrylic materials, which were mounted and sealed on a semiconductor cooling plate. This double-wall design can minimize the heat loss to the surroundings and maintain the bulk environment (locations far away from the cooling plate) in the chamber at a temperature of $20\pm1℃$ during the experimental measurements. We controlled and modulated the relative humidity ($\chi$) inside the chamber by flushing it with humid nitrogen, which was produced by mixing the streams of pure nitrogen and that of nitrogen saturated with water vapor. By adjusting the mixing ratio of these two steams (by using mass flow controllers, Figure S1), relative humidity in the range of $30\%-70\%$ has been achieved (Table S2). In the experiment, the temperature of the cooling surface was monitored by a thermocouple while a hygrometer (consisting of relative humidity & temperature sensors) was employed to detect the environmental temperature and humidity in real-time. The condensation and frosting phenomena occurring on diverse cooling surfaces were recorded with a CCD camera (U3-3060CP-M-GL, IDS Scheer, Germany) at a frame rate of $100-400 fps$ and a resolution of $1.4 \mu m/$pixel, wherein a cold light source was used for the vertical illumination. Meanwhile, the heat transfer process in the multiphase transition phenomena was monitored with an infrared camera (A655sc, FILR, USA) at a frame rate of $100 fps$ and a resolution of $33 \mu m/$pixel. To combine the optical and thermal imaging systems, a *s*ingle crystal germanium (SCG) lens, which has good reflexivity for visible light (70 %) and also owns a high infrared transmittance (approximately $95\%$ in the wavelength range of $9-12 \mu m$), was exploited in the light path (Figure S1).

The procedures of the condensation frosting experiment are as follows. A solid substrate was placed on the cooling plate in the environmental chamber, and humid nitrogen of desired gas ratio was then introduced through the inlet hole. After the temperature and relative humidity inside the chamber become stable, which generally takes $\sim30$ minutes, we began to cool the solid surface through two stages. In the first cooling stage, the temperature of the solid surface ($T_{s}$) was lowered from the environmental temperature ($T_{0}\approx20\pm1℃$) to a value below the dew point ($T_{d}=2-14℃$, depending on the relative humidity ^[^[^1^](#_ENREF_1)^]^) but above the freezing point of water ($0 ℃$), which allows for heterogeneous vapor condensation. As shown in Figure 1 of the manuscript, massive water drops nucleated and grew on the surface, and the sizes of condensate drops can be tuned by controlling the duration of surface cooling. When condensate drops of desired sizes were generated, the second cooling stage was then implemented by decreasing $T_{s}$ to the freezing temperature below $0 ℃$ ($T_{f}=-2℃--25℃$), during which condensate drops become supercooled. We generally observed the freezing of condensate drops within 30 minutes after $T_{f}$ was reached.


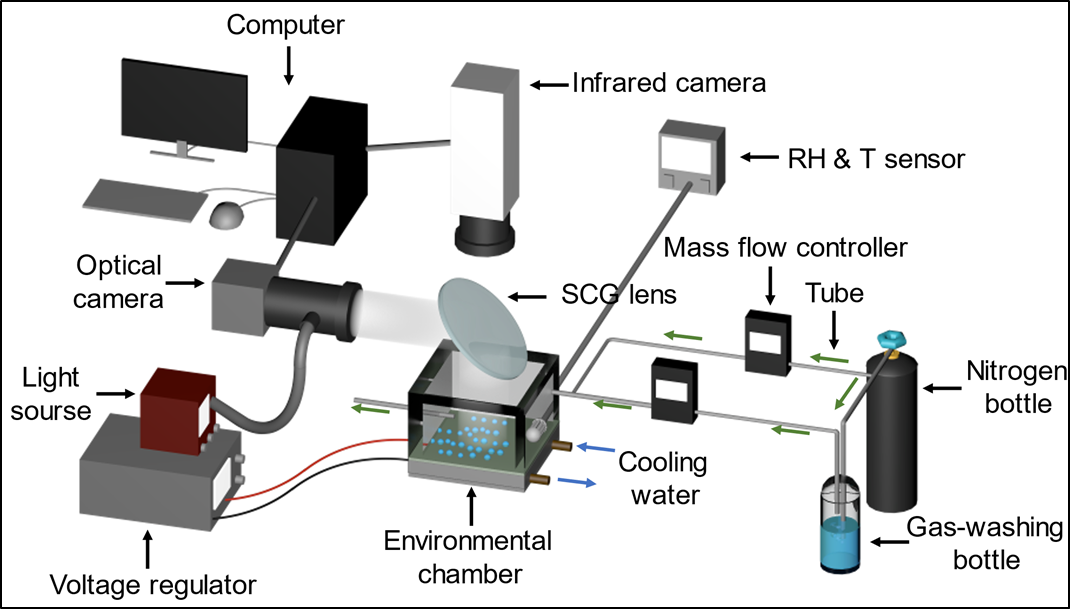


**Figure S1.** Sketch of the experimental setup.

Supplementary Section 2. Light reflection from a solid surface covered by a sessile drop

Because of their hemispherical geometry and optical transparency, condensate water drops can behave as Plano-convex lenses, which reflect and refract incident light, eventually appearing as solid black circles with a white spot at their center in the acquired optical images, as shown in Figure S2A. To get more insight into these optical effects, we numerically calculated the reflection of an incident parallel light illuminating on a solid surface covered by a water drop with $\theta_{eq}\approx120^{\circ}$, corresponding to the condensate drop in Figure S2A. Due to the symmetry of the sessile drop, two-dimensional calculation has been performed. As sketched in Figure S2B, a parallel light beam is illuminated on the drop-covered surface. While the incident light rays on the drop surface would be partially reflected back to the space and partially refracted into drop, which subsequently undergoes the reflection at the solid-liquid interface back to the drop and the refraction at the liquid-air interface back into the air, light rays vertically incident on the solid surface should be directly reflected back. In the calculation, the reflection law and Snell's Law were used to define the directions of the reflected and refracted light rays, whereas the Fresnel formula was employed to calculate their intensities.

In Figure S2C, we comparatively show the axial distribution of the relative pixel intensity value $I(x/R_{d})/I_{c}$ of the acquired drop image in the experiment (i.e. along the dotted line in Figure S2A) and of the relative light intensity $I_{r}(x/R_{d})/I_{rc}$ of the resultant reflected light from the drop-covered surface in the numerical calculation (i.e. along the dotted line in Figure S2B). Here $I(x/R_{d})$ and $I_{c}$ denote the pixel intensity values at the relative position $x/R_{d}$ and drop center ($x/R_{d}=0$) in Figure S2A, while $I_{r}(x/R_{d})$ and $I_{rc}$ denote the reflected light intensities at the relative position $x/R_{d}$ and drop center ($x/R_{d}=0$) in Figure S2B, where $x$ is the distance to the drop center and $R_{d}$ is the drop radius. Obviously, a good agreement between the experimental data and numerical calculation has been reached. Employing the calculated spatial light intensity distribution in Figure S2C, we further reproduced a two-dimensional image of the sessile drop in Figure S2D, which indeed matches the observed drop in the experiment (Figure S2A).

The light interception phenomenon discussed above also happens for condensate water droplets in the halo pattern, which makes the halo region appear dark. Moreover, the larger and the denser the condensate droplets are, the stronger the light interception is, and the darker the halo pattern is. This optical effect provides us a simple method to determine the relative sizes and density of condensate droplets during the halo pattern evolution. As an example, we calculated the average pixel intensity ($I$) in a sector region (originating from the center of the freezing drop with an angle of $10^{\circ}$) along the radial direction ($x$) of the freezing drop in Figure 2A of the manuscript during the three-stage halo pattern evolution, and relevant results are shown in Figure S2E. It is seen that upon recalent freezing, $I$ in the region beyond the drop starts to decrease and it decreases faster in the zone close to the drop, suggesting a radial decrease in the size and density of condensate droplets. During the bottom-to-top drop freezing, $I$ continues to decrease in the whole halo region, indicating that vapor condensation happens in the whole halo region. By contrast, in the halo fading stage, $I$ begins to increase and the increase is faster in zone close to the drop, implying that the evaporation of condensate droplets is stronger there, and accordingly the droplet size should be smaller.

**Figure S2.** A) Snapshot of a condensate drop sitting the Teflon-coated glass substrate ($\theta_{eq}\approx120^{\circ}$), which was acquired using the optical imaging system in Figure S1. B) Illustration of light ray behaviors after the parallel incidence to a drop-covered surface. C) Plot of the relative pixel intensity value ($I/I_{c}$) along the dashed line in A) and the relative light intensity ($I_{r}/I_{rc}$) along the dashed line in B) as a function of the relative location $x/R_{d}$. D) Reproduced image of a two-dimensional drop (top view) based on the calculated spatial light intensity distribution in C). E) Plot the average pixel intensity ($I$) in a sector region (which originates from the center of the freezing drop with an angle of $10^{\circ}$) along the radial direction of the freezing drop ($x$) in Figure 2A of the manuscript during the three-stage halo pattern evolution.

# Supplementary Section 3. Drop freezing modes in condensation frosting

In the condensation frosting experiments, we have identified two distinct modes of drop freezing: spontaneous freezing and ice-bridge-triggered freezing. While the former represents the occurrence of ice nucleation on an arbitrary drop among massive condensate drops ^[^[^2^](#_ENREF_2)^]^, as shown in Figure 1 of the manuscript and Figure S3A below, the latter refers to the freezing of a condensate drop triggered by connecting a growing ice bridge from a nearby frozen drop ^[^[^3^](#_ENREF_3)^,^ [^4^](#_ENREF_4)^]^, as displayed in Figure S3B. Figure S3C shows the zoom-in view of the growth of an ice bridge that reaches a neighboring drop and initiates the freezing in Figure S3B.

**

**

**Figure S3.** A) Selected snapshots of spontaneous drop freezing on the Teflon-coated glass substrate. B) Selected snapshots of ice-bridge-triggered drop freezing on the Teflon-coated glass substrate. C) Zoom-in view of the growth of an ice bridge from a frozen drop towards a supercooled drop marked in B). D) Plot of the statistical probability of the two drop freezing modes as a function of the freezing temperature $T_{f}$ in condensation frosting experiments on the Teflon-coated glass substrate.

A statistical analysis of the probability of observing these two freezing modes in the condensation frosting experiments on Teflon-coated glass substrates ($k\approx0.9 W/mK$ and $\theta_{eq}\approx120^{\circ}$) has been performed and the results at different freezing temperatures ($T_{f}$) are summarized in Figure S3D. Evidently, the ice-bridge-triggered freezing is always the dominant freezing mode in condensation frosting, regardless of the freezing temperature; yet, its occurrence probability slightly decreases with increasing $T_{f}$. By contrast, the spontaneous drop freezing was only observed at $T_{f}\lesssim-10 ℃$, and the lower the freezing temperature, the higher occurrence possibility would be.

# Supplementary Section 4. Observation of condensate halos on different solid surfaces

We have identified three levels of strength for condensate halos during the freezing of supercooled drops on solid substrates with different thermal conductivities and wetting properties. By analyzing the variation of the average pixel intensity value ($\bar{I}$) in a surface area of $7 \mu m\times7 \mu m$ (5 pixel $\times$ 5 pixel) adjacent to the supercooled drop ($\sim7 \mu m$ to the drop surface) during its freezing, one can find that the maximum value of the average pixel intensity $\bar{I}_{max}$ appears before drop freezing, while the average pixel intensity reaches a minimum value $\bar{I}_{min}$ when the condensate halo is completely developed. Therefore, the ratio of the minimum and maximum pixel intensity values $\bar{I}_{min}$/$\bar{I}_{max}$ stands for the contrast of the halo pattern, and thus can be used to define the strength of the halo phenomenon.

In this work, the strong halo phenomena were identified for $\bar{I}_{min}$/$\bar{I}_{max}\lesssim0.7$, for which the formation and evolution of the halo pattern can be clearly observed and analyzed via image processing, as shown in Figure S4A. Weak halo phenomena were defined by $0.7\lesssim\bar{I}_{min}$/$\bar{I}_{max}\lesssim0.9$, exhibiting poor visibility and detail ― though they can still be recognized, as shown in Figure S4B. For freezing drops with $\bar{I}_{min}$/$\bar{I}_{max}\gtrsim0.9$, the variation of the pixel intensity value in their nearby region was very small during drop freezing (Figure S4C), and thus they are classified as "no halo". Figure S4D summarizes the experimental conditions for the appearance of these three classes of condensate halos in condensation frosting. It is obvious that condensate halos can only be observed on solid substrates with sufficiently low thermal conductivities ($k\lesssim10 W/mK$) and surface wetting properties (${60^{\circ}\lesssim\theta}_{\mathrm{eq}}\lesssim150^{\circ}$), and the more hydrophobic the surface, the clearer the halo pattern is.

In principle, condenstate halos or equivalently vapor condensation should occur during drop freezing if two necessary conditions are saftisfied: establishing a supersaturated water vapor around the freezing drop (i.e., making the solid-vapor system metastable) and overcoming the nucleation energy barrier (or equivalently the nucleus size should exceed a critical value). Practically, however, one can condensate halo patterns only if both the spatial and temporal resolutions of the optical imaging system are sufficiently high as they might be formed by very small droplets and last only for very short time. Nevertheless, in a recent study Shang and co-workers have also reported that there may exist a critical drop size of $\sim17 \mu m$, above which the formation of condensate halos can be triggered during its freezing on the superhydrophobic surface.^[^[^5^](#_ENREF_5)^]^ To validate this finding on the Teflon-coated hydrophobic surface, additional experiments have been performed to visualize the freezing of very small water droplets in condensation frosting. It turned out that within the spatial and temporal resolution limits of our optical imaging system, condensate halo evolutions can still be observed around freezing drops with radii down to $9 \mu m$, as shown in Figure S4D.

**Figure S4.** A) Selected snapshots of strong halo phenomena. B) Selected snapshots of weak halo phenomena. C) Selected snapshots of freezing drops without condensate halos. D) Phase diagram of condensate halos in condensation frosting on solid surfaces with diverse thermal conductivities and wetting properties. E) Top: selected snapshots of the freezing of a $9 \mu m$-radius condensate drop on the Teflon-coated hydrophobic surface. Bottom: the pixel intensity value along the radial coordinate defined in the top image during the halo pattern evolution (i.e., formation, growth and fading).

# **Supplementary Section 5. Halo dynamics and the associated temperature evolution in the ice-bridge-triggered drop freezing**

Figure S5A displays a sequence of halo dynamics and the associated temperature evolution in the ice-bridge-triggered freezing of a supercooled condensate drop on the Teflon-coated glass substrate at $T_{f}=-14.5 ℃$ and $\chi\approx60 \%$. Similar to that in the spontaneous drop freezing, condensate halo consisting of tiny drops instantly forms at its full size on the solid surface in the short-time recalescent freezing, which is identified by the decrease of the pixel intensity values in the optical images and the increase of surface temperature in the thermographic images on the drop and its nearby region, as shown in Figure S5B. In the subsequent bottom-to-top drop freezing, the continuous existence of the warm vapor around the partially-frozen drop results in further vapor condensation on the solid surface, and the as-formed condensate drops grow in their sizes, making the halo pattern darker. This halo growth stage ends when the whole drop was frozen, along with the decay of the warm vapor filed. Then, the frozen drop starts to harvest water vapor that evaporates from condensate drops in the halo fence region, and the halo pattern completely disappears within a few seconds.





**Figure S5.** A) Selected optical and thermographic images showing the ice-bridge-triggered drop freezing process on the Teflon-coated hydrophobic surface at $T_{f}\approx-14.5 ℃$. B) Temporal evolution of the pixel intensity value $I$ and surface temperature $T_{S}$ at the center of the freezing drop and two nearby locations denoted in A). The right figures are zoom-in view of the initial halo formation stage.

# Supplementary Section 6. Detection of gas temperature and humidity around freezing drops on solid surfaces of different thermal conductivities

To get more insight into the effect of substrate thermal conductivity on the formation of condensation halos, we simultaneously measured the temperature ($T$) and humidity ($\chi^{'}$) in the surrounding gas during the freezing of millimeter-sized supercooled water drops (with a volume of $4.2 \mu L$) at $T_{f}=-16 ℃$ on glass ($k=0.93 W\cdot m^{-1}\cdot K^{-1}$), steel ($k=10 W\cdot m^{-1}\cdot K^{-1}$), silicon ($k=160 W\cdot m^{-1}\cdot K^{-1}$), and copper ($k=400 W\cdot m^{-1}\cdot K^{-1}$) substrates with similar wetting property ($\theta_{eq}=110\pm5^{\circ}$). Note that condensate halos can only be observed on the glass (strong) and steel (weak) substrate while no halos would form on the silicon and copper substrates during drop freezing (Figure S4D). As sketched in Figure S6A, the gas humidity was detected by a humidity sensor locating at $\sim1.2 mm$ from the drop surface, while the temperature field around the drop was monitored by an infrared camera. The initial temperature and relative humidity were respectively set at $20\pm1℃$ and of $55\pm3\%$, which are in the parameter range of our condensation frosting experiments.

In Figure S6C, we comparatively show selected thermographic images of freezing drops on these four substrates, while the corresponding variations of the relative humidity $\chi^{'}/\chi_{0}^{'}$ are displayed in Figure S6D, where $\chi_{0}^{'}$ is the initial humidity before freezing. Upon recalescent freezing, a significant increase of the drop temperature from the supercooling value ($\sim-16 ℃$) to the melting point of ice ($\sim0 ℃$) has been identified on all drops, and simultaneously $\chi^{'}/\chi_{0}^{'}$ starts to increase. This finding suggests that the sudden temperature rise causes the explosive drop evaporation, which sends out water vapor to the surroundings. Despite the water vapor field was established around all drops throughout their whole freezing process, condensate halos only form on substrates with low thermal conductivities, i.e., $k\lesssim10 W\cdot m^{-1}\cdot K^{-1}$. A detailed comparison of the acquired thermographic images reveals that along with the sudden rise of drop temperature, there is also a rise of the temperature of the surrounding gas. This phenomenon can be more clearly seen from the temporal variation of the temperature difference ($\Delta T$) before and during the drop freezing in Figure S6C, where representative thermographic images are also displayed. However, on the glass and steel substrates, the temperature rise of the surrounding gas of freezing drops on glass and steel substrates (where condensate halos can form during drop freezing) are much higher than that on silicon and copper substrates (where halo patterns cannot form); similar behaviors are also identified for the variation of the relative humidity during the freezing. Based on the above findings, we can explicitly draw a conclusion that, apart from the buildup of the vapor field by the freezing-induced vapor diffusion, establishing a “high” temperature field is the other crucial condition to trigger the halo formation in drop freezing.





**Figure S6.** A) Sketch of the experimental setup to simultaneously measure the temperature and relative humidity around freezing water drops (left) on four hydrophobic substrates with similar wetting property but different thermal conductivity, and a snapshot showing the drop in the experiment (right). B) Selected thermographic images showing the drop freezing processes on four different solid substrates. C) Plot of the temperature rise $\Delta T$ in the surrounding gas close to the drop as a function of the freezing time $t$ (left), and thermographic images (right) showing the temperature difference field around the freezing drop at the time of reaching the maximum $\Delta T$ as indicated in the top figure. D) Temporal evolution of the humidity ratio $\chi^{'}/\chi_{0}^{'}$ during freezing.


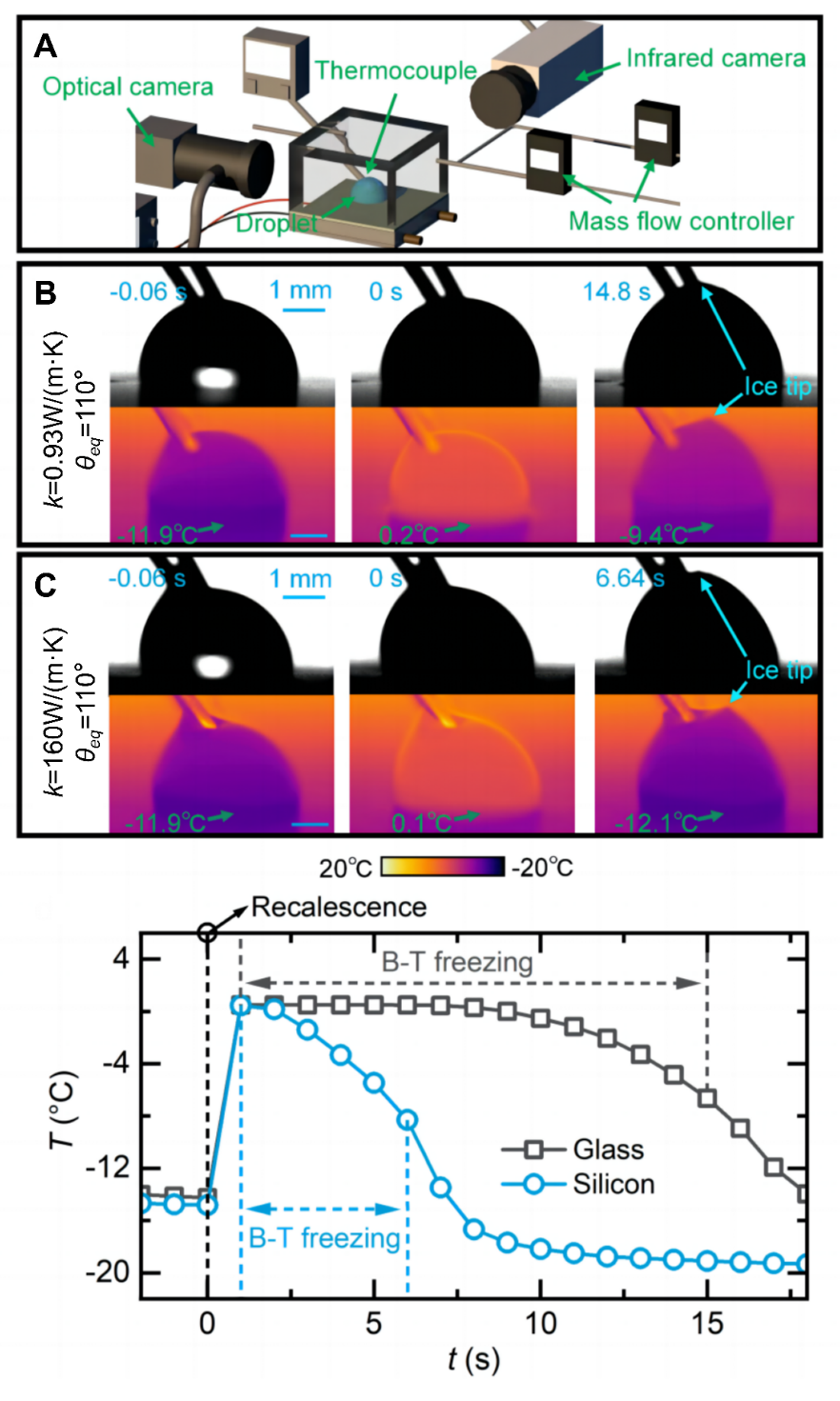


**Figure S7.** A) Sketch of the experimental setup to simultaneously measure the temperature inside and outside freezing water drops (left). B) Selected optical and thermographic images showing the drop freezing processes on the glass substrate. C) Selected optical and thermographic images showing the drop freezing processes on the silicon substrate. D) Temporal evolution of the water temperature inside the drop during freezing.

Now, an obvious question to ask is the following: What causes the different temperature rise in the surrounding gas of diverse freezing drops. Given the fact that the only different condition for the above drop freezing experiments is the substrate thermal conductivity, we would explain it as the result of distinct heat transfer process on these substrates. Upon recalescence, the released latent heat from the liquid solidification is partly adsorbed by the drop, causing its temperature rise, and partly would be transferred to the underlying cooling stage through the substrate. However, due to the finite thermal conductivity of solid materials, the rate of heat transfer is limited; in other words, the heat transfer through the solid substrates is insufficient to remove all the released latent apart from that absorbed by the drop. As a result, some heat would be taken away by the surrounding gas, rising its temperature. Because the recalescent freezing takes very short period [$O(10 ms)$], it is impossible to detect this transient heat transfer process. Nevertheless, we have managed to probe the heat transfer in the drop during the bottom-to-top freezing, which lasts for several hundreds of milliseconds. As illustrated in Figure S7A, a thermocouple temperature sensor was inserted into the drop to measure the temperature variation in the liquid during the freezing. Meanwhile, the infrared camera was still employed to monitor the temperature variation on drop surface, and selected snapshots are shown in Figure S7B. Figure S7C reports the temporal variation of water temperature inside freezing drops on the glass and silicon substrates. Evidently, the decrease of the liquid temperature is much slower on the glass substrate than that on the silicon substrate, which demonstrates the limited heat transfer on low thermal conductive substrates and thus indirectly verifies our hypothesis.

# Supplementary Section 7. Evolution of the established vapor field during the bottom-to-top (B-T) drop freezing

Since the B-T freezing time or equivalently the halo growth time $t_{g}$ is much longer than the characteristic vapor diffusion time $\tau_{D}$ (e.g., for the freezing drop with radius $R_{d}\approx150 \mu m$ in Figure 2 of the manuscript, $t_{g}\approx0.5 s$ while $\tau_{D}=R_{d}^{2}/D_{v}\approx1.2\times{10}^{-3} \mathrm{ms}$), a quasistatic approximation can thus be employed to describe the vapor concentration. That is, at any given time of the freezing, the vapor diffusion would be modeled using the following equations,

$J=-D_{v}\nabla C$ (1)

$\nabla J=0$ (2)

where $J$, $D_{v}$, and $C$ are the evaporation flux, vapor diffusion coefficient, and vapor concentration, respectively. By solving the above equations with the commercial software — COMSOL Multiphysics 5.4, we can obtain the vapor concentration field around a freezing drop and further estimate the size of the halo pattern.

As an example, we calculated the vapor field evolution during the B-T freezing of an individual $0.4 mm$-radius water drop on the Teflon-coated glass substrate at $T_{f}=-17 ℃$, which was experimentally observed using the high-speed camera. Before freezing, the vapor concentration at the droplet surface and in the far field (i.e. the location at a distance of $5R_{d}$ from the droplet) are defined as $C_{s}$ and $C_{s}\cdot\chi$, respectively, as illustrated in Figure S8A, where $C_{s}$ represents the saturation concentration of vapor at the ambient temperature. The vapor diffusion coefficient $D_{v}$ is also set as the value at ambient temperature. Upon recalescent freezing, ice crystals would grow inside the supercooled drop and ~20% of liquid water turns into ice; that is, the drop becomes a mixture of water and ice, and is partially frozen. Accordingly, the temperature of the drop instantaneously increases to $0 ℃$, and in principle its vapor concentration should be adjusted to the saturation concentration of vapor at $0 ℃$, which we denote as ${C'}_{s}$. Nevertheless, previous work has demonstrated that by defining the vapor concentration as $0.78{C'}_{s}$,^[[6](#_ENREF_6)]^ a good agreement between the theoretical calculation and experimental result can be achieved; therefore, this concentration value was used in the following calculation. Once B-T freezing is initated, the bottom of the partially-frozen drop would start to freeze completely, and the drop has a composite configuration: its bottom part is completely frozen and becomes iced, while its top part is still partially-frozen. With the ongoing of B-T freezing, the height of ice $H_{ice}$ increases with time (the black line in Fig. S8D), and the volume of the partially-frozen water at the drop top decreases. To calculate the vapor field around the composite drop during the B-T freezing, the boundary condition at the ice surface needs to be given. It is known that the vapor pressure at the ice surface is approximately zero, and thus would harvest water molecules from its surrounding environment. That is, ice is a hygroscopic material, which has a negative evaporation flux near its surface. However, for freezing drops in condensation frosting, water vapor in the region just above the cooling solid surface has been mostly condensed as water drops, and the corresponding vapor pressure is very low. As a result, the vapor pressure gradient around the frozen drop and thus the evaporation flux should be rather low. For the sake of simplicity, the evaporation flux can be assumed to be zero (Fig. S8B), as suggested by Poulikakos and co-workers in Ref. [[6](#_ENREF_6)].

In Figure S8C, we show the representative vapor concentration fields around the supercooled drop before and during its freezing ($t=0-1.0 s$). Clearly, the vapor concentration field around a supercooled drop is very weak because of its low temperature t. However, once the freezing is triggered, a strong vapor filed would be established due to the increase of the drop temperature, and the vapor field gradually shrinks with the propagation of the icing front from drop bottom to top. Making a reasonable assumption that condensate halo forms on the solid surface region, above which the vapor concentration is equal or higher than the saturation concentration at the supercooled temperature, the halo radial extent $R_{hm}$ was calculated. As shown in Figure S8C and Figure S8D, $R_{hm}$ is on the order of $R_{d}$ and it decreases with the freezing time would be expected. This simulation result agrees well with that of the previous work.^[^[^6^](#_ENREF_6)^]^


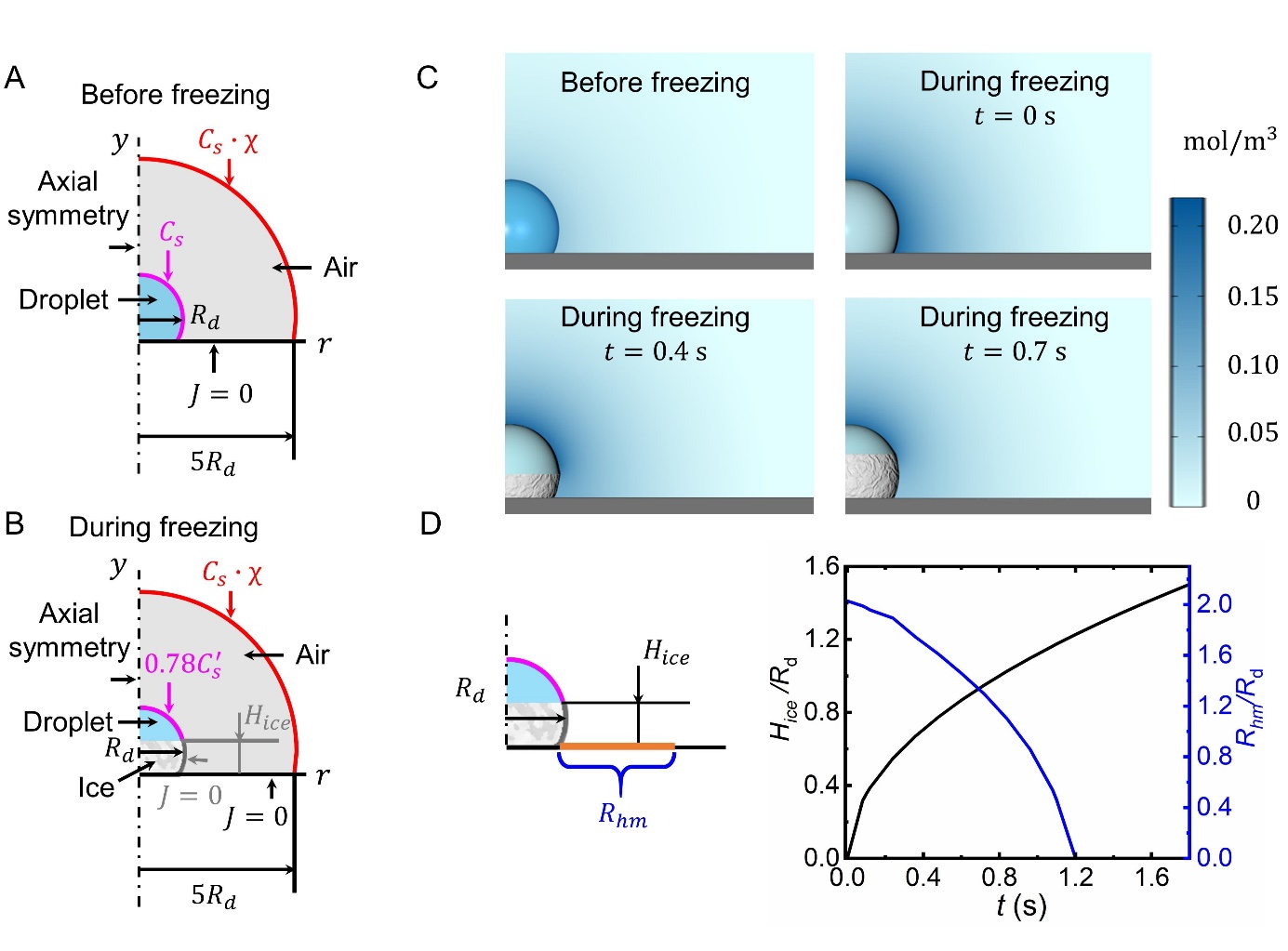


**Figure S8.** A) Sketch of the supercooled drop before freezing. B) Sketch of the freezing drop. C) Vapor concentration fields around the supercooled drop before and during its freezing. D) Temporal evolution of the normalized height ($H_{ice}/R_{d}$, the black line) of the freezing front on the drop in the experiment, and the normalized radial extent ($R_{hm}/R_{d}$, the blue line) of the condensate halo in the calculation.

# Supplementary Tables

**Table S1.** Stream rates of pure nitrogen and water vapor saturated nitrogen to achieve different relative humidity in the environmental chamber in Figure S1.

| Stream rate of pure nitrogen ($\mathrm{cm}^{3}/min)$ | Stream rate of water vapor saturated nitrogen ($\mathrm{cm}^{3}/min)$ | The resultant relative humidity in the environmental chamber (%) |
| --- | --- | --- |
| 0 | 350 | 74 |
| 0 | 90 | 68 |
| 0 | 0 | 62 |
| 145 | 0 | 50 |
| 400 | 0 | 38 |

**Table S2.** The Péclet numbers for mass (${Pe}_{m}=lu/D_{v}$) and heat (${Pe}_{h}=lu/\alpha$) transfer in the experiments, where $l$ is the characteristic length and should be on the order of the halo radial extension $\Delta\tilde{R}_{hm}$, $u$ is the velocity of the flushing humid nitrogen, $D_{v}$ is the diffusion coefficient of water molecules in the vapor phase and $\alpha$ is the thermal diffusivity.

| Nitrogen flow velocity $u$ ($\mathrm{mm}/s)$ | Characteristic length $l$ ($mm)$ | Thermal diffusivity $\alpha$ ($\mathrm{mm}^{2}/s$) | Diffusion coefficient $D_{v}$ ($\mathrm{mm}^{2}/s$) | Péclet number for heat transfer ${Pe}_{m}$ | Péclet number on mass transfer ${Pe}_{h}$ |
| --- | --- | --- | --- | --- | --- |
| 1.22 | 0.1 | 0.39 | 24 | 0.31 | 0.005 |
| 1.22 | 0.1 | 4.2 | 24 | 0.03 | 0.005 |
| 1.22 | 0.1 | 90 | 24 | 0.001 | 0.005 |
| 1.22 | 0.1 | 117 | 24 | 0.001 | 0.005 |

**Table S3.** Thermal and wetting properties of diverse substrates used in the experiments.

| Substrate number | Materials | Thermal Conductivity $k$ ($Wm^{-1}K^{-1}$) | Equilibrium contact angle of $4 \mu L$ water drop $\theta_{eq}$ ($^{\circ}$) |
| --- | --- | --- | --- |
| 1 | Pure glass | 0.93 | $31\pm4^{\circ}$ |
| 2 | Glass silanized by 3-aminopropyltriethoxysilane | 0.93 | $62\pm3^{\circ}$ |
| 3 | Glass silanized by 1,1,1,3,3,3-Hexamethyldisilazane | 0.93 | $88\pm1^{\circ}$ |
| 4 | Glass silanized by 1H,1H,2H,2H-perfluorodecyltriethoxysilane | 0.93 | $117\pm2^{\circ}$ |
| 5 | Glass covered by Teflon | 0.93 | $120\pm1^{\circ}$ |
| 6 | Glass covered by hydrophobic nanoparticles | 0.93 | $151\pm1^{\circ}$ |
| 7 | Pure steel | 10 | $80\pm1^{\circ}$ |
| 8 | Steel silanized by 1,1,1,3,3,3-Hexamethyldisilazane | 10 | $88\pm1^{\circ}$ |
| 9 | Steel silanized by 1H,1H,2H,2H-perfluorodecyltriethoxysilane | 10 | $106\pm1^{\circ}$ |
| 10 | Steel covered by hydrophobic nanoparticles | 10 | $152\pm1^{\circ}$ |
| 11 | Pure silicon | 160 | $60\pm2^{\circ}$ |
| 12 | Silicon silanized by 1,1,1,3,3,3-Hexamethyldisilazane | 160 | $87\pm1^{\circ}$ |
| 13 | Silicon silanized by 1H,1H,2H,2H-perfluorodecyltriethoxysilane | 160 | $113\pm1^{\circ}$ |
| 14 | Silicon covered by hydrophobic nanoparticles | 160 | $150\pm1^{\circ}$ |
| 15 | Pure copper | 400 | $76\pm2^{\circ}$ |
| 16 | Copper silanized by 3-aminopropyltriethoxysilane |  | $61\pm5^{\circ}$ |
| 17 | Copper silanized by 1H,1H,2H,2H-perfluorodecyltriethoxysilane | 400 | $103\pm1^{\circ}$ |
| 18 | Copper covered by hydrophobic nanoparticles | 400 | $151\pm1^{\circ}$ |

# Table S4. Roughness of diverse solid substrates.

| Substrates | Roughness (nm) | Topographic AFM images |
| --- | --- | --- |
| Pure glass substrates and silanized glass substrates (i.e., substrates 1-5 in Table S2) | 0.64$\pm$0.03 | 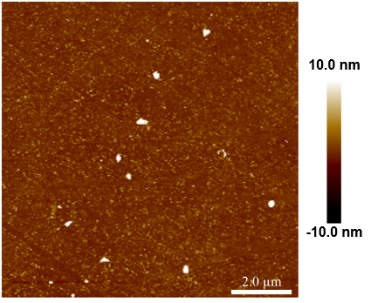 |
| Pure steel substrates and silanized steel substrates (i.e., substrates 7-9 in Table S2) | 2.15$\pm$0.92 | 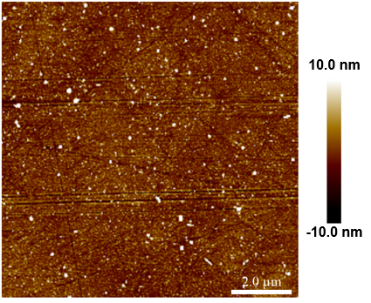 |
| Pure silicon substrates and silanized silicon substrates (i.e., substrates 11-13 in Table S2) | 0.17$\pm$0.01 | 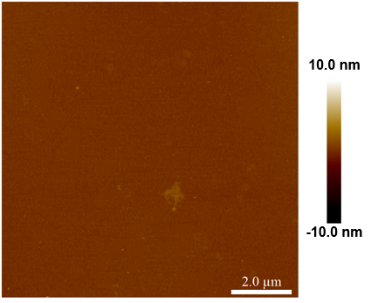 |
| Pure copper substrates and silanized copper substrates (i.e., substrates 15-17 in Table S2) | 45.93$\pm$19.83 | 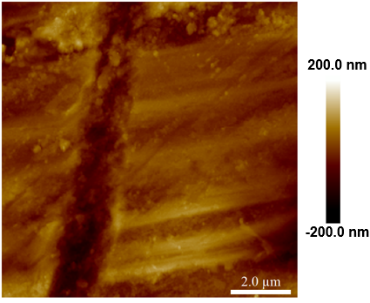 |
| Substrates covered by hydrophobic nanoparticles (i.e., substrates 6, 10, 14 and 18 in Table S2) | 58.53$\pm$27.59 | 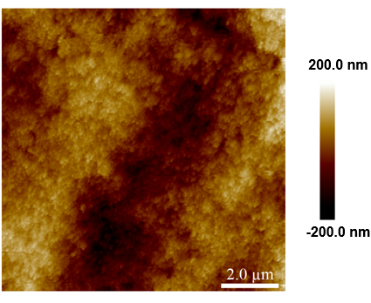 |

# Supplementary Movies

**Supplementary Movie 1** A representative condensation frosting process on a glass substrate coated with a $1 \mu m$-thickness Teflon. The thermal conductivity ($k$) of the substrate is $0.9 Wm^{-1}K^{-1}$ and the equilibrium contact angle ($\theta_{eq}$) of $4 \mu L$ water drops on the surface is $120^{\circ}$. The movie was recorded at 20 fps.

**Supplementary Movie 2** Freezing of condensate water drops on a Teflon-coated glass substrate with $k \approx0.9 Wm^{-1}K^{-1}$ and $\theta_{eq}\approx120^{\circ}$ at $T_{f}=-13.1 ℃$. The initial relative humidity ($\chi$) for the condensate frosting experiment was $60 \%$. The optical and thermographic movies were recorded at 100 fps.

**Supplementary Movie 3** Spontaneous freezing of a condensate water drop on a Teflon-coated glass substrate with $k \approx0.9 Wm^{-1}K^{-1}$ and $\theta_{eq}\approx120^{\circ}$ at $T_{f}=-14.5 ℃$. The initial relative humidity ($\chi$) for the condensate frosting experiment was $60 \%$. The optical and thermographic movies were recorded at 100 fps.

**Supplementary Movie 4** Ice-bridge triggered freezing of a condensate water drop on a Teflon-coated glass substrate with $k \approx0.9 Wm^{-1}K^{-1}$ and $\theta_{eq}\approx120^{\circ}$ at $T_{f}=-15.1 ℃$. The initial relative humidity ($\chi$) for the condensate frosting experiment was $60 \%$. The optical and thermographic movies were recorded at 100 fps.

**Supplementary Movie 5** Recalescent freezing of a $3.3 \mu L$ water drop on a Teflon-coated glass substrate with $k \approx0.9 Wm^{-1}K^{-1}$ and $\theta_{eq}\approx120^{\circ}$ at $T_{f}=-13.2 ℃$ and $\chi=55 \%$. The movie was recorded at 5000 fps.

**Supplementary Movie 6** A representative frost propagation among four supercooled condensate drops on a Teflon-coated glass substrate with at $T_{f}=-14.4 ℃$ and $\chi=65\%$. The movie was recorded at 100 fps.

# References

[1] M.G. Lawrence, *Bull. Am. Meteorol. Soc.*, **2005**, 86 225.

[2] C. Gurganus, A.B. Kostinski, R.A. Shaw, *J. Phys. Chem. Lett.*, **2011**, 2 1449.

[3] J. Guadarrama-Cetina, A. Mongruel, W. Gonzalez-Vinas, et al., *Europhys. Lett.*, **2013**, 101 16009.

[4] J. Petit, E. Bonaccurso, *Langmuir*, **2014**, 30 1160.

[5] Y.H. Shang, D. Li, H.Z. Liu, *Phys. Fluids*, **2024**, 36.

[6] S. Jung, M.K. Tiwari, D. Poulikakos, *Proc. Natl. Acad. Sci. USA*, **2012**, 109 16073.
